# Supplementary material for: Distinct Gut Microbial Enterotypes and Functional Dynamics in Wild Striped Field Mice (Apodemus agrarius) across Diverse Populations
Source: Microorganisms. 2024 Mar 28;12(4):671. doi: 10.3390/microorganisms12040671 (PMC11052172; doi:10.3390/microorganisms12040671)
Supplement: Supplementary file 1 [file microorganisms-12-00671-s001.zip › Supplementary Figures.pdf]

Supplementary Information for:

Distinct Gut Microbial Enterotypes and Functional Dynamics in Wild Striped Field Mice (*Apodemus agrarius*) Across Diverse Populations

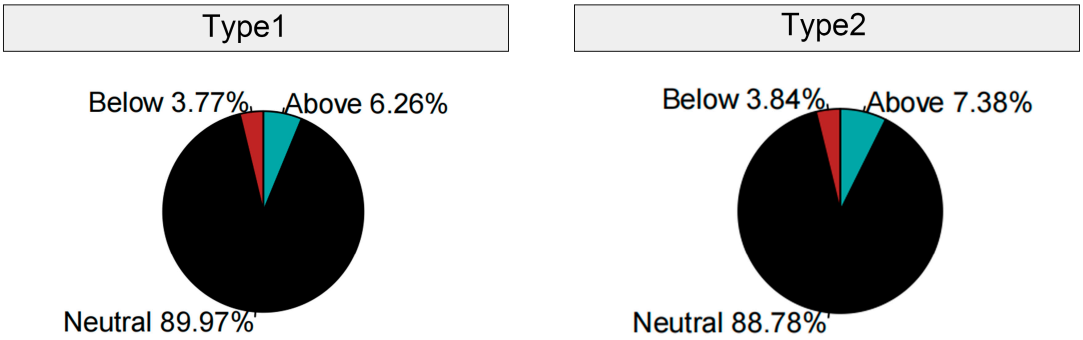

Figure S1 The degree of fitting of different intestinal types of ASVs in the neutral model. Percentage represents the number of ASVs within and outside the 95% confidence interval of the neutral model prediction.

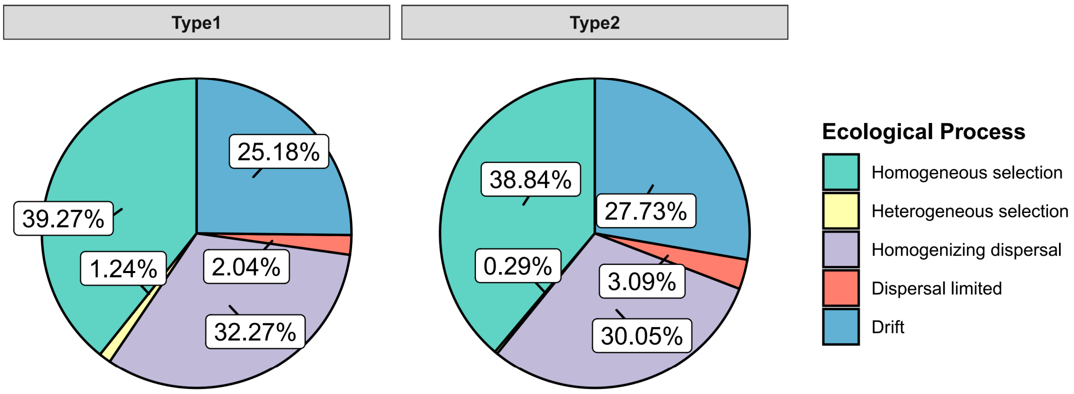

Figure S2 Contributions of deterministic (| βNTI | ≥ 2) and stochastic processes (| βNTI | < 2) on gut microbiota community assembly in different enterotype of *A. agrarius*.
